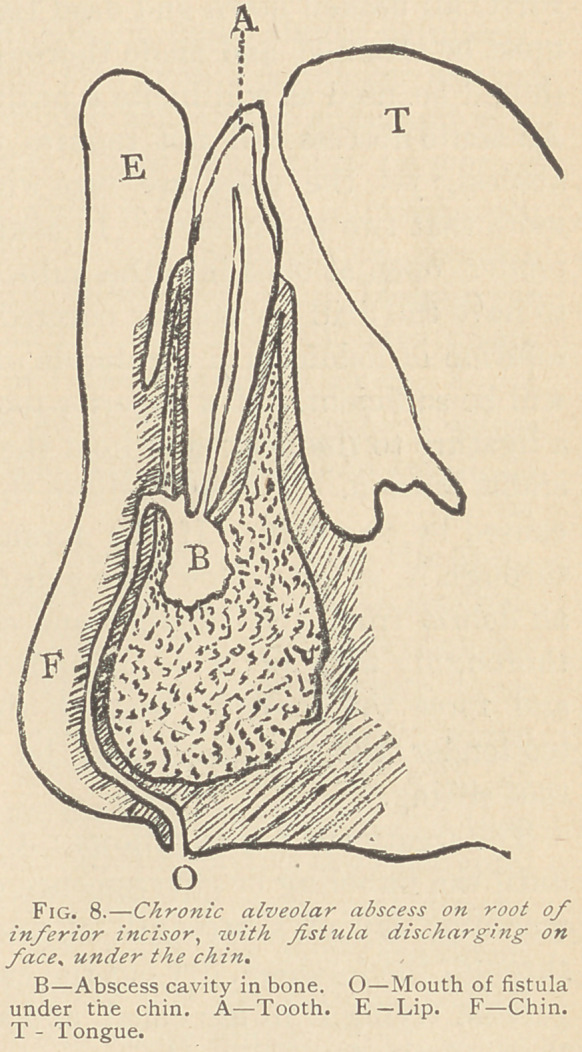# Alveolar Abscess

**Published:** 1887-07

**Authors:** Geo. A. Maxfield

**Affiliations:** Holyoke, Mass.


### ALVEOLAR ABSCESS.

### GROWTH, DEVELOPMENT AND TREATMENT.

Read before the Vermont State Dental Society at the Annual Meeting, held in Burlington, Vt., March 17, 1887.

BY GEO. A. MAXFIELD, D. D. S., HOLYOKE, MASS.

The word Abscess is defined as a collection of pus in an abnormal cavity, the result of a morbid process. When found at the root of a tooth, having commenced at the apex, it is called an Alveolar Abscess ;* the acute inflammation of the pericementum being the morbid cause by which it is induced.

Acute pericementitis proceeds from direct local irritation, and this arises, with but few exceptions, from one of two causes—an inflamed pulp, or a dead pulp.

Acute pericementitis may or may not be induced by an inflamed pulp ; it cannot be induced by a dead pulp until air has been admitted to the pulp chamber.

The theory that a dead pulp always putrefies, decomposing into gaseous elements which cause pressure upon the parts about the foramen, producing an irritation from which pericementitis and abscess result, is an erroneous one. That putrefaction of the dead pulp often, and I might almost say always, produces acute

* It is well known that abscesses occur on other parts of the root than the apex, but the originating causes being different from those of Alveolar Abscess, I do not consider them in this paper.

pericementitis, I firmly believe, but putrefaction of a dead pulp cannot occur till there has been exposure to the atmosphere.

"From the experiments of noted scientists, especially that of Pasteur, it is shown that putrefaction is not occasioned by the chemical action of oxygen or any other gas, but is a species of fermentation, analogous to that of sugar under the influence of a growing yeast-plant, being brought about by a development of microscopic organisms, the germs of which, from their extreme minuteness, float in abundance in the air as constituents of its dust." * Therefore, when there is an abscess at the apex of the root of a tooth, where the pulp chamber is a closed cavity so that air is unable to penetrate, the inflammation of the pericementum must have been induced by the inflamed pulp, and the death of the pulp is the result of the pericementitis and not the cause of it. A pulp may be inflamed and die, and yet not exert any deleterious influence upon the pericementum, and a tooth with a dead pulp may remain in a healthy condition for years, or even through life. Again, an inflamed pulp may induce a chronic or sub-acute pericementitis, causing the death of the pulp, and the tooth may remain in this condition, that is, with a dead pulp and with chronic pericementitis, for years and no abscess result.

The causes of an inflamed pulp I will not attempt to consider in this paper. They are apparent to every observing practitioner. But just here it will be useful briefly to consider the anatomical structure of the pericemental membrane ; it is undoubtedly a continuance of the periosteum that everywhere covers bone, except where covered with cartilage, with the exception that in the tooth socket there is some difference in its structure, as it has additional functions to perform ; it is of a double surface, that is, it has to supply nutriment from both sides, to the bone and to the tooth ; it also has to serve as a cushion for the tooth, enabling it to resist the blows of mastication. It is a dense, fibrous membrane, richly supplied with blood vessels and nerves, and is much thicker at the apex of the root.

It is supplied with blood at the apex from the artery that supplies the pulp, and these arteries anastomose freely with the arteries in the alveolus that supply the gums, also with the arteries in the periosteum at the borders of the socket. The nerves also proceed from

* Holmes' Surgery, vol. 3, page 375.

the same sources as the blood supply, or more largely from the periosteum at the borders of the socket, and in this membrane that the nerves of touch for the tooth are located.

Another question to consider at this point is, what are the causes of suppuration? One of the most important questions before the scientific world to-day is, "can there be suppuration without the presence of micro-organisms?" * For many years it has been conceded that bacteria is one cause of suppuration, but within the past year it has been demonstrated, through rigorous experiments by some of the leading investigators, that there is no suppuration whatever without the action of living bacteria. In the *Medical Record* of Dec. 25, 1866, Dr. H. Knapp, of New York, published a valuable paper on "Fermentation, Putrefaction and Suppuration," in which he gives the various experiments with the results obtained, which were entered upon to determine the above question, and from this paper I will quote at some length. He says: "the same species of bacteria will produce suppuration as well as putrefaction. The pyogenic fungi will, for instance, curdle and decompose milk, and from putrefying substances bacteria are obtained that have a strong pyogenic action. Suppurating substances are of the same nature as the putrescible. The difference only is, that suppuration has relation to living, putrefaction to dead, substances or tissues. Both are split into simpler and analogous compounds."

He studies this subject under the following heads, "Does traumatism of any kind produce suppuration? Do foreign bodies occasion a formation of pus? Are there any kinds of chemical agents that cause suppuration?" In answer to the first question, he says, "I performed all the operations that are practiced on the eye, on the one side of a rabbit with sterilized instruments in an aseptic way; on the other side the wound was contaminated with an emulsion of a pure culture of some pyogenic fungus. All the former healed by first intention, the latter suppurated with the regularity of a chemical experiment. One of the fundamental observations that led to the introduction of antiseptic surgery, was the fact that simple fractures heal without suppuration, but there is an occasional exception to this rule. In the exceptional cases of suppuration after a simple fracture, a focus of suppuration somewhere else in the body has either been discovered, or its presence

* Editorial in INDEPENDENT PRACTITIONER for February, 1887.

can be assumed with certainty ; the experimental proof of this fact has been furnished of late by Beeker and F. Krause, who observed that simple fractures in healthy animals regularly healed by first intention, but just as regularly suppurated when pyogenic microbes were injected into a vein of the ear, and he says, "this galaxy of facts furnishes indisputable evidence that mere traumatism of whatever kind can never cause suppuration.

FIG. 1 —*Commencement of alveolar abscess.*

A—Superior incisor tooth. B—Abscess cavity in the bone. E—Lip. F—Hard palate. D—Floor of the nostril.

As to the second question, Do foreign bodies, as such, cause suppuration ? he says, "Theo. Leber, and others, have experimentally studied and brought it to a certain final solution. Leber states that indifferent non-oxidizable foreign bodies aseptically introduced into the tissues or cavities of the human body caused no inflammation, in particular no suppuration. I have introduced a number of foreign bodies, for instance, pieces of a hair-pin, aseptically into the anterior chamber of the right eye of a rabbit. The hair-pin was old, rusty and dirty. It was not cleansed, but before its introduction it was brought to a glow. A small piece of the periphery of the cornea was cauterized with a glowing strabismus hook, pierced with a sterilized small knife ; the pin was introduced with an aseptic little forceps, pushed more deeply into the anterior chamber and the wound canal again sealed with the glowing hook. The foreign bodies caused no suppuration ; they either lay free on the iris or were surrounded with a delicate, apparently fibrous exudation. A similar piece of the same hair-pin had been brought to a glow and introduced in the same way into the other eye, with the only difference, that before the introduction it had been dipped into an emulsion of *staphylococcus pyogenes albus,* with the result, that the most violent phlegmon broke out in this eye within the first twenty-four hours, and destroyed the globe completely.

As to the third question—Do chemical agents cause suppuration without the intervention of microbes?—he says: "This question has been answered positively by all but a few recent investigators. Baumgarten, Theo. Leber, Uskoff, Orthmann, Councilmann, Rosenbach, Passet and others, assert, having convinced themselves, that suppuration is caused by certain chemical agents, for instance, mercury, oil of cantharides, petroleum, turpentine, and above all, croton oil, even if aseptically introduced. Four recent observers, however, contest this assertion on the strength of new and more rigorous experiments. The leading investigator of the four is J. Straus, who has, for the first time, used a perfect aseptic method. A sterilized glass tube, tapering to a point, on one side was closed with a sterilized cotton plug, and the other filled with sterilized croton oil, and the point sealed up. He sterilized the skin of the animal by singeing it with Paquelin's cautery, stabbed it with a sterilized knife, introduced the thin end of the tube, broke off its point, through the cotton plug blew the oil out into the sub-cutaneous tissue, withdrew the tube and sealed the wound-canal by burning its orifice with the cautery. Among eighteen injections of turpentine, thirteen did not produce suppuration; of five injections of croton oil, none suppurated; of two injections of mercury, none suppurated. When suppuration ensued he found cocci in the pus.

FIG. 2.—*Acute alveolar abscess on root of superior incisor, pointing on the gum.*

A—Tooth. B—Abscess cavity in the bone. E—Lip. D—Floor of the nostril. F—Hard palate.

E. Scheuerlen, of Berlin, in his experiments used small glass tubes, sterilized for half an hour in a Koch steam sterilizer, then by means of a canula needle and a small piston thrust under the skin of rabbits. Before the operation the skin was carefully shorn and disinfected with a one to one thousand corrosive sublimate solution, and after the introduction the little tube lay in the sub-cutaneous tissue, about ten millimeters distant from the puncture of the skin. The region of the puncture was covered with a thick layer of iodoform collodium. He used a dozen irritating substances in the tubes, among them turpentine and croton oil. A week or ten days after the introduction of the little tubes, when they lay free from irritation under the skin, and the small wound was perfectly healed, they were broken. A hard swelling occurred around them, but suppuration in only one of his thirty-two experiments (croton oil), in which a puriform exudation extended from the skin all along the stab-wound. In this case the sterilization and healing had been imperfect, and bacteria, which were found in the purulent exudation, must have penetrated into the wound. Two others, Klemperer and Dr. J. A. Rugs, made similar experiments with like results, and the conclusion they all reached from their experiments was that bacteria are the cause of every suppuration."

FIG. 3.—*Acute alveolar abscess with pocket of pus between the periosteum and bone.*

B—Abscess cavity in the bone. C—Pus cavity between periosteum and the bone. A—Tooth. E—Lip. F—Hard palate. D—Floor of nostril.

Dr. Knapp experimented in a similar manner, with like results, and in conclusion he says: "They furnish, it seems to me, sufficient evidence of the truth of the proposition, that suppuration in every case depends on the action of microbes. If, on one hand, traumatism of any kind, if foreign bodies, if the most irritant chemical agents, if anything you may imagine is not of itself capable of producing suppuration; if on the other hand, the addition of pyogenic microbes to any irritating substance or wound, or any lesion whatsoever, under proper conditions, produces suppurations without fail, we are certainly justified in ascribing to pyogenic germs the causative action in the formation of pus. What is pus? 'An albuminous, non-coagulable fluid, containing multitudes of leucocytes.' What is suppuration? 'The splitting of living nitrogenous tissue into simpler compounds, through the influence of certain bacteria.' In this way the parallelism of the three processes—fermentation, putrefaction and suppuration—is established."

I have quoted Dr. Knapp's paper at such length, because it sustains me in the theory I have maintained for some time, that a dead pulp is not of itself the cause of an alveolar abscess. That these abscesses always begin at the apex of the root is not to my mind any proof that the dead pulp is the exciting cause, for at this point the tissue of the pericementum is the thickest, and it is also the center from which radiates its rich plexus of nerves and blood vessels, and because of its bony confinement an irritation is not so easily overcome. We say "the death of the pulp is the result of external violence to the tooth." It is simply one of the results of the inflammation of the pericementum, induced by the shock. Starting, then, with an inflamed pulp in a closed cavity where air has not been able to obtain entrance, the inflammation may extend to the pericementum by the continuity of the parts, the congestion and swelling of the pericemental tissue causing a stricture of the vessels entering the apical foramen of the tooth, and thus literally strangulating the pulp to death.

FIG. 4.—*Acute alveolar abscess, with pocket of pus underneath the periosteum of the hard palate.*

B—Abscess cavity in the bone. C—Pus cavity between the periosteum and the bone. A—Tooth. E—Lip. F—Hard palate. D—Floor of the nostril.

According to the stage which the inflammation has now attained, if pyogenic germs are absent, the inflammation may quickly subside and the dead pulp may pass into a semi-fluid condition, or dry down and become, as we say, "mummified." If the inflammation is severe, it may be controlled and its duration shortened by the use of prophylactic and sedative measures. That the inflammation in some of these cases continues to suppuration I do not deny, but I do not believe it is possible until the pyogenic germs have in some manner obtained entrance. Where the inflammation of the pulp occurs as the result of an exposure, the death of the pulp and the entrance of the germs is easily explained. That in these cases an abscess does not always result from the death of the pulp, is easily explained by the supposition that, through some means, the foramen closed up, preventing the entrance of the germs. The death of the pulp may have been so gradual that inflammation was not excited and the foramen closed with a healthy eschar.

As soon as the germs have obtained entrance to the seat of inflammation at the apex, the tissues rapidly disintegrate and pus begins to form. (See Fig. 1.*) The pain which was severe and steady is now more intense, and accompanied with a throbbing which is characteristic. Inflammation extends over the periosteum while the gum over the tooth also becomes congested and swollen. The pressure of the pus in the pent-up cavity causes more or less absorption of the surrounding bone. It is the universal rule that pus will burrow in the direction of the least resistance, and the external lamina of bone, being more dense and hard than the interior, it often happens that there is considerable destruction of bone about the apex, quite a cavity being thus formed, before an opening is made. The sack that is found adhering to the root when an abscessed root is extracted, is the disorganized tissue. It generally adheres to the root because the surrounding bone, being the softest, it is more easily absorbed than the root. The membrane surrounding the disorganized tissue, forming the sack called the "pyogenic membrane," that was formerly thought to secrete pus, is simply the line of demarkation between the dead and living tissue.

FIG. 5.—*Chronic alveolar abscess on root of superior incisor, with fistula discharging into the nose.*

A—Tooth. B—Abscess cavity in the bone. O—Mouth of fistula. D—Floor of the nostril. E—Lip. F—Hard palate.

As soon as the pus makes an exit through the bone into the soft tissues, the pain begins to abate and the features often become much swollen. (Fig. 2.) As soon as a fistula is formed and an outlet furnished the pus, the pain decreases and the swelling rapidly subsides. Concerning that condition in which the pus, instead of penetrating into the soft tissues, separates the periosteum from the

* I am indebted to the kindness of Dr. W. C. Barrett for the loan of the charts from which these cuts were made, he having used them at Hartford, Conn., June, 1886, to illustrate the same subject in an address before the Conn. Valley Dental Society.

bone and forms a cavity for itself between the two (Figs. 3 and 4.), Dr. G. V. Black,* says: "This is the form of abscess that is most likely to be attended with necrosis of portions of bone, and for this reason should receive prompt attention for the purpose of preventing or limiting this very unfavorable result. This seems to occur mostly in those cases in which the inflammation has run high, and in which there has been or exists at the time of the escape of the pus from the bone a very considerable inflammation of its substance and of its periosteum, by which the layer of osteoblasts have become so softened that they are readily separated from the bone beneath. In this condition of things, the pus in making its escape from the bone, instead of penetrating the overlaying tissues raises the periosteum in the same manner as in sub-periosteal inflammation. In this way separation of the periosteum from the bone over a considerable surface occasionally occurs, and if the parts are suffered to remain in this condition for a considerable time, necrosis more or less extensive will result. If, on the other hand, the pus be promptly discharged, so that the periosteum may be again brought in contact with the parts from which it was separated, not much harm will follow; it will readily become reattached and the parts will heal without difficulty. Separation of the periosteum is to be suspected when the tumor of the gum is broad and comparatively soft. This form of abscess, when left to itself, is prone to discharge at the gingival margin, after having separated the periosteum from the outer wall of the alveolar process. In this condition the only blood supply that this portion of the process can obtain is that which may come from the other side of the tooth, through the anastomosis of the arterial branches in the peridental membrane, already in a more or less inflamed condition, or through

FIG. 6.—*Chronic alveolar abscess on root of superior incisor, with fistula discharging on the gum.*

B—Abscess cavity in the bone. O—Mouth of fistula. A—Tooth. E—Lip. F—Hard palate. D—Floor of the nostril.

* American System of Dentistry, Vol. 1, page 932.

the Haversian canals of the septum of the alveolar process between the teeth. This will at once be seen, in the inflamed condition of the parts, to be a very precarious supply, and as a result of this condition necrosis of the alveolar plates overlaying the root affected and those immediately adjacent, is very liable to occur."

FIG. 7.—*Chronic alveolar abscess on root of inferior incisor, with abscess cavity passing through the body of the bone and discharging on the face, under the chin.*

B—Abscess cavity. O—Mouth of fistula under the chin.

A—Tooth. E—Lip. F—Chin. T—Tongue.

When these abscesses occur they are liable to point on the face, and the more so if they occur on the inferior jaw. Occasionally these abscesses discharge through other mucus membranes than those of the mouth; those from the superior molars discharging into the antrum of Highmore; those from the superior incisor tooth into the nose (Fig. 5,) and sometimes burrowing beneath the tissues and discharging behind the soft palate into the throat. Sometimes the pus from an abscess on the superior molars will burrow through the muscles of the cheek and appear just below the malar prominence, or it may burrow along underneath the muscle and appear below the jaw, as if from a lower molar. This is generally the result of the acute form (See Figs. 3 and 4), but may be the result of a chronic abscess.

As soon as an opening is made and the pus discharged, the abscess assumes a chronic form. (Fig. 6.) The cases in which an alveolar abscess heals of its own accord after it discharges are rare, for this reason: "Pus continues to form as long as a dead part remains in contact with a living seat." * The dead pulp in this case

* Garretson's Oral Surgery, page 775.

may contribute towards the continuance of the abscess, though the necrosed bone which is found in most of these cases is a sufficient, and often the only irritant that remains to keep up a constant discharge.

Sometimes the opening closes up permanently, and there remains a small amount of pus and disorganized tissue in the apical space,. which forms what is termed a blind abscess. (Fig. 1.). This condition may continue for years, but is liable to have periodic fits of soreness similar to chronic pericementitis. On the inferior jaw, the closing of the fistula in the mouth is very liable to occur, and the pus, simply from the force of gravitation, will burrow in the soft tissues and sometimes through the bone, pointing, perhaps, under the chin and opening another fitsula (Figs. 7 and 8), or perhaps burrowing further down beneath the tissues and appearing on the neck or shoulder.

FIG. 8.—*Chronic alveolar abscess on root of inferior incisor, with fistula discharging on face, under the chin.*

B—Abscess cavity in bone. O—Mouth of fistula under the chin. A—Tooth. E—Lip. F—Chin. T - Tongue.

The treatment varies somewhat according to the condition of the abscess. Generally the removal of the irritant and the placing of the parts in an aseptic condition is all that is necessary. If it is not deemed best to attempt to save the tooth, it should be promptly extracted, no matter at what stage it presents itself, and unless there is considerable necrosed bone, the abscess will probably heal at once. I find that the very erroneous opinion prevails among some practitioners that it is unsafe to extract a tooth during the formation and progress of an abscess. As this savors of the age when barbers and blacksmiths were the only dentists, I fail to understand how any intelligent practitioner can harbor such an opinion. The cases are very rare, however, that extraction of the tooth need be resorted to as a means of cure.

The treatment for the cure of alveolar abscess is very simple. The knowledge of the fact that suppuration is always caused by the presence of bacteria, indicates the line along which it must proceed. First, all broken down and dead tissues must be removed, the parts must be cleansed and made thoroughly aseptic, then such remedies should be used as will incite a healthy action in the parts involved. An acute abscess will not require as much treatment as a chronic abscess, yet the cases are rare where even chronic cases require more that two treatments. In acute cases, if the tooth is not too tender, open at once into the pulp chamber, taking the precaution to have the drill constantly covered with an aseptic fluid—either a solution of iodoform or bichloride of mercury, one to one thousand, will be sufficient. If a superior tooth, and it is quite tender, apply a ligature to the neck and hold the ends in the hand, with considerable tension. This will relieve the pressure on the pericementum caused by the drill. If the foramen is closed, pass a small drill through it, so as to allow free exit of the discharges, first removing all debris from the chamber and canal. Then wash out the canal thoroughly with peroxide of hydrogen; if possible, use a syringe and force the peroxide through the foramen. If the tooth is not too tender, the treatment may be continued and the root filled at that sitting.*

The treatment † is as follows: Continue the use of the peroxide until the bubbling or effervescence ceases; then apply bichloride of mercury, say of a strength of one to one thousand, and force it through the foramen, dry out the canal and fit to it a cone of gutta-percha, trying it in the canal, and so cut that it will come a little short of the foramen. As soon as the cone is ready apply a saturated solution of iodoform in extract of eucalyptol, pumping it well up into the canal and through the foramen; then pump a solution of gutta-percha into the canal, dip the cone into the

---

* In these cases I always treat and fill at a single sitting, unless the tooth is too sore to permit the necessary manipulation.

† This treatment is practically the same as that first advocated by Dr. C. T. Stockwell, in a paper read before this society last year. Of sixty-two cases which I have treated in this way the past year, the only failures—that is where the fistula had not healed within three weeks after the operation—were two old chronic cases, one a superior incisor of eight years' standing, the other a first inferior molar of over six years'.

same solution and press it home to the foramen. The remainder of the filling can be finished at any time. If the cleansing has been thoroughly done, the abscess will give no more trouble.

If the tooth is too sore to admit of an opening being made into the pulp chamber, make an opening from the outside through the gum to the apex of the root. This can be done almost painlessly, by the use of cocaine before lancing the gum. A quicker method is to apply a little of the crystal carbolic acid to the gum, and with a lance cut well down to the bone, which must then be pierced with a small drill. Paint well the gums around the affected tooth with a mixture of equal parts of tincture aconite root, tincture iodine, and chloroform,* which will relieve the pain. Dismiss the patient for twenty-four hours, when the soreness will have disappeared and it can then be treated as I have indicated above.

In these cases it is sometimes advisable to prescribe a saline cathartic, also bromide of potassium, say sixty grains in two doses on retiring for the night.

Take particular care in opening up the canals to make the openings sufficiently large that free access to the canals will be secured.

For Chronic Alveolar Abscess: First open up the pulp chamber and canal and thoroughly cleanse. If it is of long standing, there will undoubtedly be some necrosed bone in the apical cavity, which must be removed. Enlarge the fistulous opening, and with an engine bur cut out all the dead bone, wash out the cavity, and then proceed as with an acute abscess, only after applying the eucalyptol pack the canal with cotton saturated with the same, and dismiss the patient for from four to seven days, when the same treatment may be renewed. If there is still considerable pus formed, treat it as before, and let it stand another week before filling.

The cases that require more extensive treatment than this are exceptional ones. For these the treatment must vary according to the complication that presents itself. Occasionally, in acute abscesses we must resort to constitutional as well as local treatment, and these must be met as the symptoms arise. To give them the consideration necessary would unduly lengthen this paper.

---

* "According to Waller, who has made very careful experiments, it has been ascertained that alkaloids dissolved in chloroform are readily transferred through the skin into the blood, and produce characteristic phenomena, while alcoholic and aqueous solutions are either not at all, or very slowly absorbed."

## Figures and Tables

**Fig. 1 f1:**
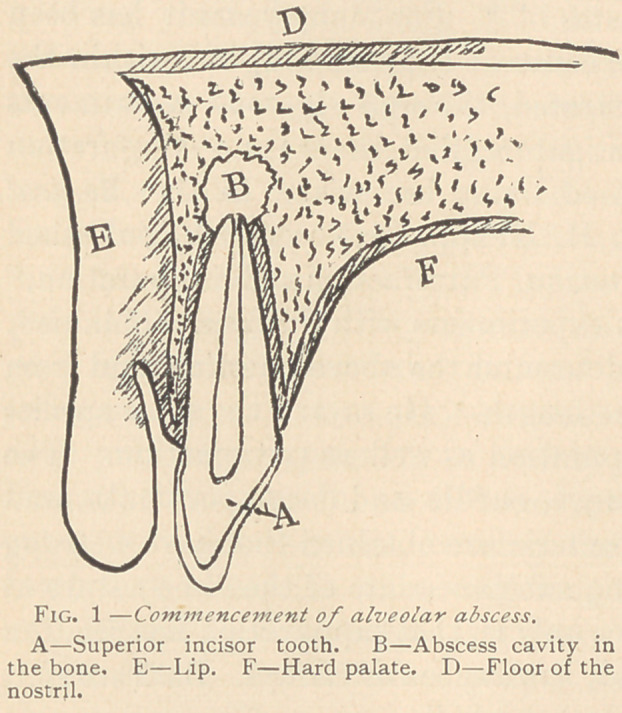


**Fig. 2. f2:**
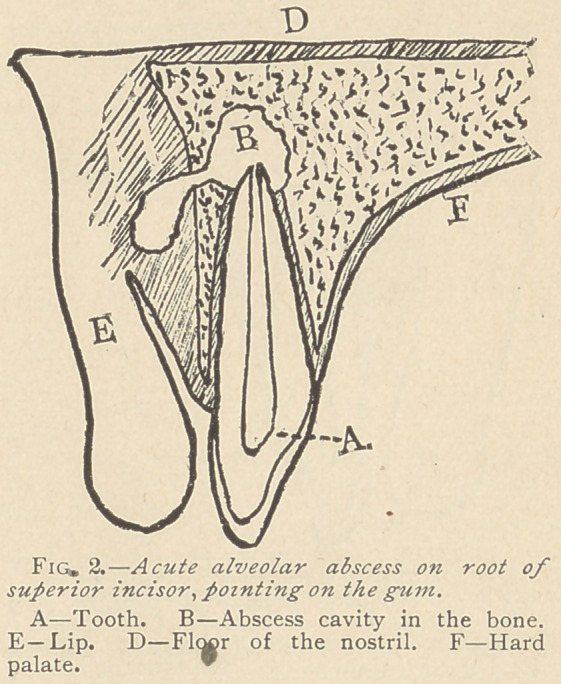


**Fig. 3. f3:**
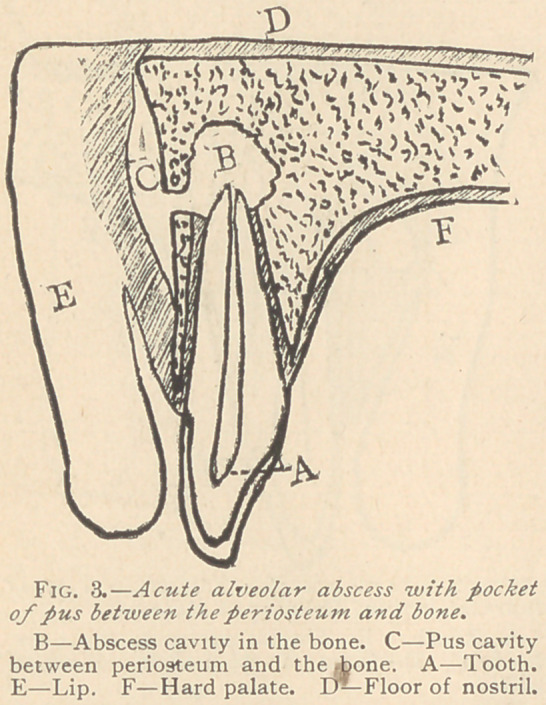


**Fig. 4. f4:**
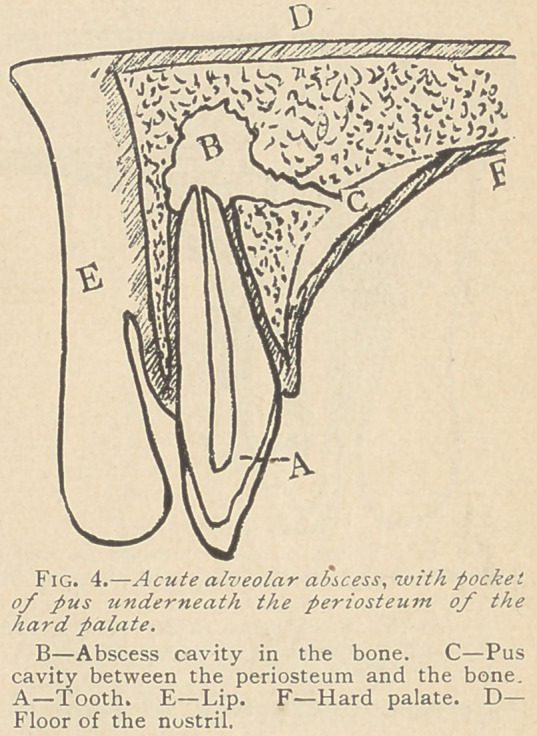


**Fig. 5. f5:**
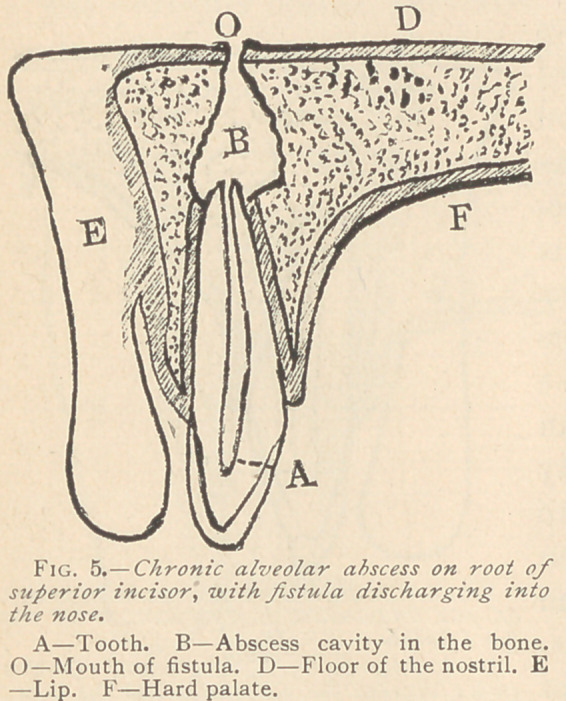


**Fig. 6. f6:**
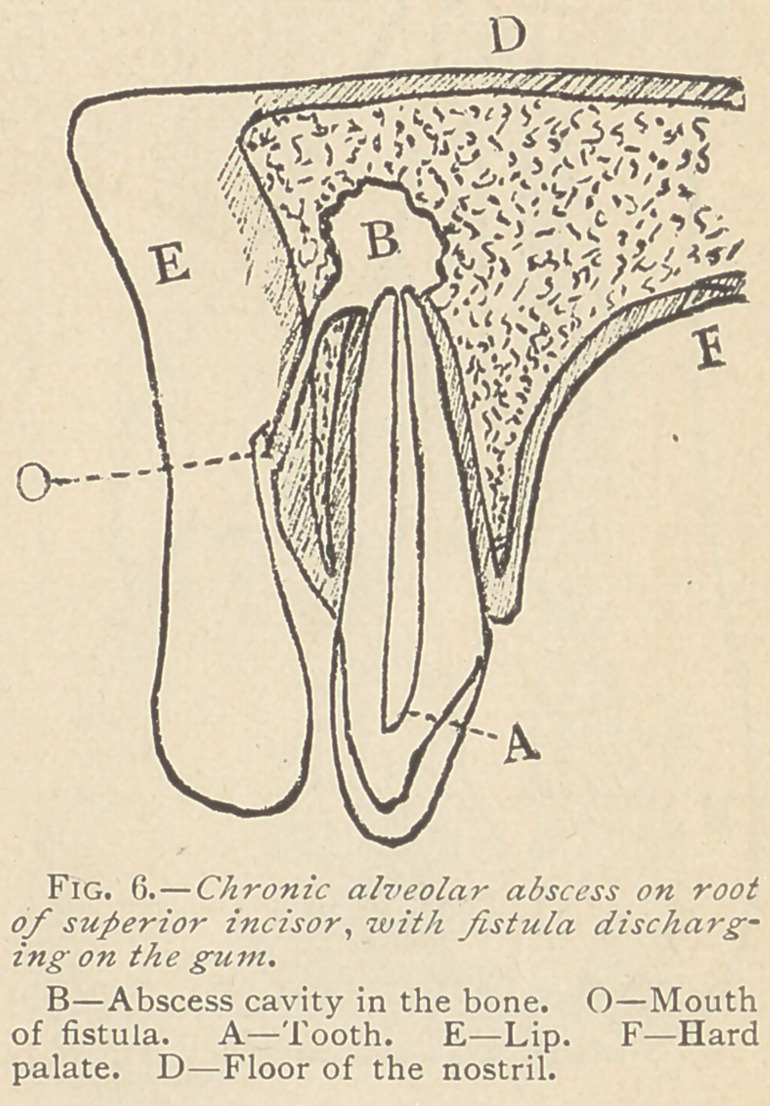


**Fig. 7. f7:**
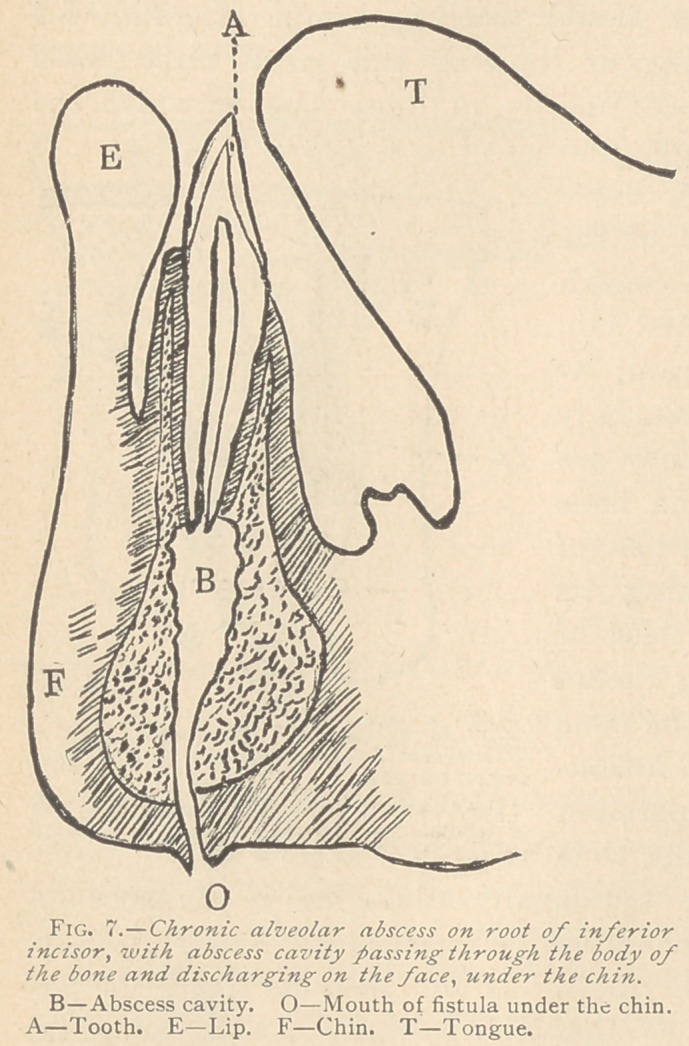


**Fig. 8. f8:**